# The impact of city-wide deployment of *Wolbachia*-carrying mosquitoes on arboviral disease incidence in Medellín and Bello, Colombia: study protocol for an interrupted time-series analysis and a test-negative design study

**DOI:** 10.12688/f1000research.19858.1

**Published:** 2019-08-01

**Authors:** Ivan D. Velez, Eduardo Santacruz, Simon C. Kutcher, Sandra L. Duque, Alexander Uribe, Jovany Barajas, Sandra Gonzalez, Ana Cristina Patino, Lina Zuluaga, Luis Martínez, María Camila Mejia, María Patricia Arbelaez, Henry Pulido, Nicholas P. Jewell, Scott L. O'Neill, Cameron P. Simmons, Katherine L. Anders, Stephanie K. Tanamas

**Affiliations:** 1 World Mosquito Program, Universidad de Antioquia, Medellin, Colombia; 2 World Mosquito Program, Institute of Vector Borne Disease, Monash University, Melbourne, VIC, Australia; 3 Secretariat of Health, Bello, Colombia; 4 Division of Epidemiology and Biostatistics, School of Public Health, University of California, Berkeley, Berkeley, CA, USA; 5 Centre for Statistical Methodology, London School of Hygiene & Tropical Medicine, London, UK

**Keywords:** Wolbachia, dengue, chikungunya, Zika, vector-borne disease, disease surveillance, interrupted time series, Colombia

## Abstract

**Background:** Dengue, chikungunya and Zika are viral infections transmitted by *Aedes aegypti* mosquitoes, and present major public health challenges in tropical regions. Traditional vector control methods have been ineffective at halting disease transmission. The World Mosquito Program has developed a novel approach to arbovirus control using *Ae. aegypti *stably transfected with the *Wolbachia* bacterium, which have significantly reduced ability to transmit dengue, Zika and chikungunya in laboratory experiments. Field releases in eight countries have demonstrated *Wolbachia* establishment in local *Ae. aegypti* populations.

**Methods:** We describe a pragmatic approach to measuring the epidemiological impact of city-wide *Wolbachia* deployments in Bello and Medellín, Colombia. First, an interrupted time-series analysis will compare the incidence of dengue, chikungunya and Zika case notifications before and after *Wolbachia* releases, across the two municipalities. Second, a prospective case-control study using a test-negative design will be conducted in one quadrant of Medellín. Three of the six contiguous release zones in the case-control area were allocated to receive the first *Wolbachia* deployments in the city and three to be treated last, approximating a parallel two-arm trial for the >12-month period during which *Wolbachia* exposure remains discordant. Allocation, although non-random, aimed to maximise balance between arms in historical dengue incidence and demographics. Arboviral disease cases and arbovirus-negative controls will be enrolled concurrently from febrile patients presenting to primary care, with case/control status classified retrospectively following laboratory diagnostic testing. Intervention effect is estimated from an aggregate odds ratio comparing *Wolbachia*-exposure odds among test-positive cases versus test-negative controls.

**Discussion:** The study findings will add to an accumulating body of evidence from global field sites on the efficacy of the *Wolbachia* method in reducing arboviral disease incidence, and can inform decisions on wider public health implementation of this intervention in the Americas and beyond.

**Trial registration: **ClinicalTrials.gov: NCT03631719. Registered on 15 August 2018.

## Abbreviations

BG trap: BioGents Sentinel trap; CI: cytoplasmic incompatibility; ELISA: enzyme-linked immunosorbent assay; IRB: Institutional Review Board; PECET: Programa de Estudio y Control de Enfermedades Tropicales; qPCR: qualitative polymerase chain reaction; SAE: serious adverse event; WEI: *Wolbachia* exposure index; WHO: World Health Organisation; wMel: *Wolbachia pipientis;* WMP: World Mosquito Program

## Background

Dengue is a major public health challenge in tropical regions, with 50 – 100 million symptomatic cases estimated to occur each year ^1, 2^. The World Health Organisation (WHO) cites a 30-fold increase in global incidence during the past 50 years ^1^, and among endemic regions the greatest relative increase in dengue disease burden over the past two decades has been seen in Latin America ^2^. The primary vector for dengue, the *Aedes aegypti* mosquito, also transmits the chikungunya and Zika viruses. Chikungunya emerged in epidemic fashion in several Indian Ocean islands in 2004 before spreading to southern Europe and South and South East Asia, then in 2013 re-emerged in epidemics in the Caribbean and several Latin American countries ^3^. Following Zika virus outbreaks in the Western Pacific in 2013 and in Latin America in 2015 ^4^, it was declared a public health emergency of international concern by the WHO in 2016 ^5^ as evidence accumulated that congenital Zika virus infections can result in severe outcomes including foetal death and severe microcephaly. No specific treatment for dengue, chikungunya or Zika currently exists. Although a vaccine against dengue (Sanofi Dengvaxia ^®^) was licensed in 2015, the WHO recommends vaccination only in persons with proven past dengue infection ^6, 7^. While efforts to develop a safe and effective vaccine continue, the WHO has emphasised the need for innovations in vector control to achieve reductions in dengue virus transmission and disease burden ^8^. The evidence base for the effectiveness of commonly used vector control interventions is limited, with few having been rigorously evaluated against a clinical disease endpoint ^9^. This highlights a vital need for carefully designed studies to evaluate vector control methods for arboviral and other vector-borne diseases ^10^.

The World Mosquito Program (WMP; formerly the Eliminate Dengue Program) is an international research collaboration that is delivering a paradigm shift in the control of arboviral diseases transmitted by *Ae. aegypti* mosquitoes. Our method utilises *Wolbachia*, obligate intracellular endosymbionts that are common in insect species ^11– 14^ but were not present in *Ae. aegypti* mosquitoes until they were stably transinfected in the laboratory. In insects, *Wolbachia* is maternally inherited and manipulates insect reproduction to favour its own population dissemination via cytoplasmic incompatibility (CI). Strikingly, the presence of *Wolbachia* in *Ae. aegypti* mosquitoes reduces their ability to transmit viruses including dengue, Zika, chikungunya, and yellow fever ^15– 17^. Introgression of *Wolbachia* into wild *Ae. aegypti* populations is thus expected to severely reduce the vectorial capacity of local mosquito populations to transmit these arboviral infections. WMP’s field teams release male and female *Wolbachia-*infected *Ae. aegypti* as eggs or adults over a number of weeks. These mosquitoes then breed with the wild mosquito population and over time, through the actions of CI, the prevalence of *Wolbachia* in the local mosquito population increases, until such time as the majority of mosquitoes in the area carry *Wolbachia*. The WMP has demonstrated reduced vector competence in *Wolbachia*-infected mosquitoes obtained from the field, using human dengue viremic blood and a novel read-out to measure infectious mosquito saliva ^18^. *Wolbachia* viral interference effects were found for all four DENV serotypes, resulting in estimated reductions of 66–75% in the basic reproduction number R _0_ for DENV-1-4. Reductions of this magnitude are predicted to result in local elimination of DENV transmission in most epidemiological settings ^18^.

Colombia, located in the northwestern region of South America, is home to one-third of the population of Latin America. The *Ae. aegypti* mosquito is highly prevalent and dengue is endemic. In 2010 Colombia recorded its largest dengue outbreak with more than 150,000 confirmed cases, 217 deaths, and simultaneous circulation of all four dengue serotypes ^19^. The first autochthonous chikungunya case was detected in Colombia in September 2014 ^20, 21^ and the first case of Zika in October 2015 ^22^. Since then, numerous cases of both have been reported in the country.

The protocol presented in the current paper describes a pragmatic approach to measuring the efficacy of large-scale *Wolbachia* deployments in reducing the burden of arboviral diseases, in the municipalities of Medellín and Bello in northwestern Colombia, which have urban populations of 2.2 million in an area of ~100km ^2^ and 476,000 in ~20km ^2^, respectively. Staged deployment at the city-wide scale and within a relatively short time frame was favoured over a randomised controlled trial or other randomised design both by funders and local stakeholders. This was driven by what was, at the time of project conception, an urgent need for novel scalable strategies to combat the threat of Zika, and also a desire for the flexibility to optimise methods for scaled deployment under operational conditions, rather than the more restrictive implementation required for a formal randomised controlled trial. The proposed strategy for evaluating the impact of these staged deployments on the incidence of arboviral disease is a combination of an interrupted time-series analysis of notified arboviral disease incidence in all of Medellín and Bello, together with a more rigorous test-negative design study implemented in a sub-section of the Medellín municipal area. The primary aim of the study is to investigate whether large-scale deployments of *Wolbachia*-infected *Ae. aegypti* mosquitoes in Medellín and Bello, Colombia, lead to a measurable reduction in arboviral disease incidence.

## Methods

**Figure 1.**
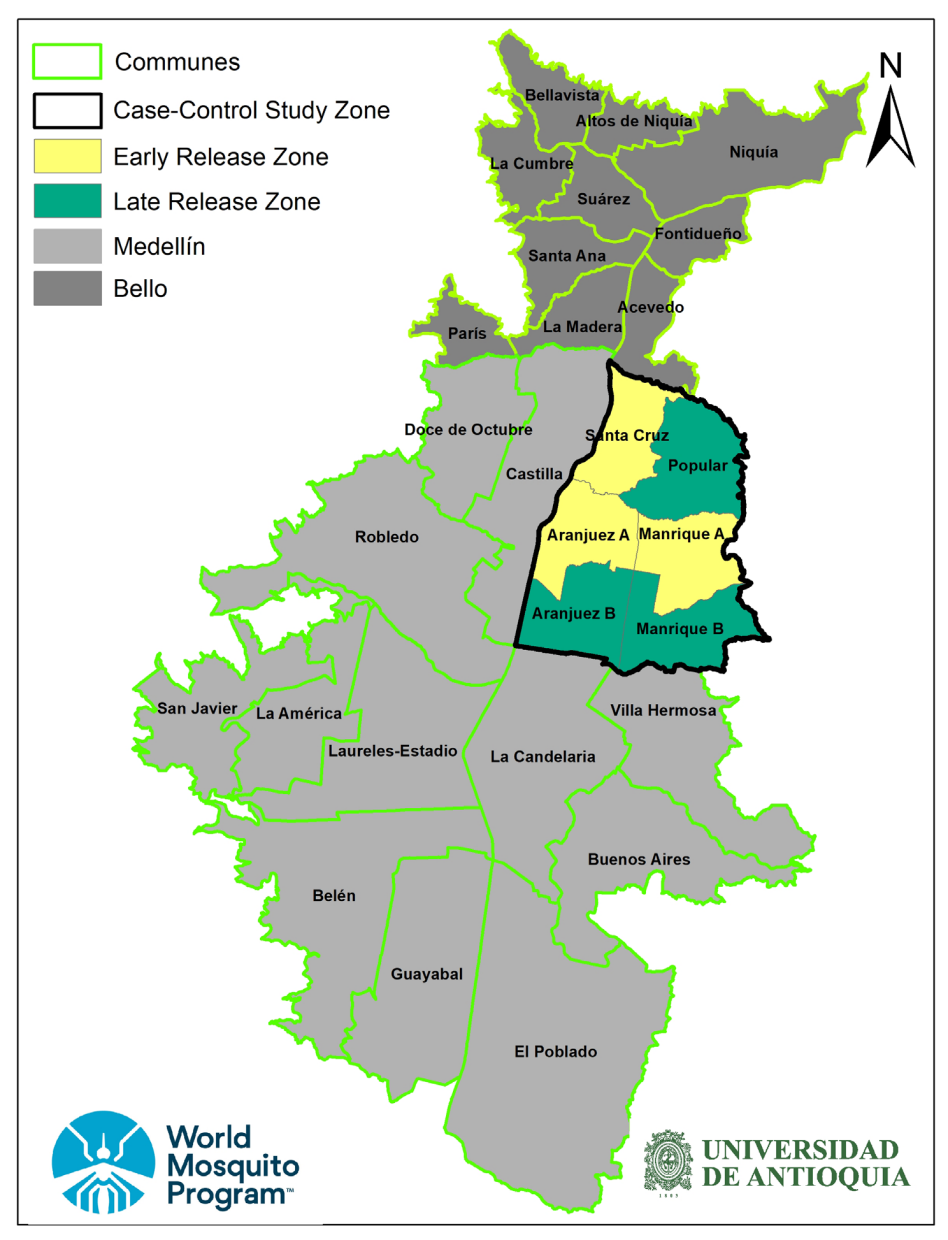
Deployment of *Wolbachia* across Medellín and Bello, combining pragmatic staged deployment (grey) with a test-negative design study in a focused study area of ‘early’ (yellow) and ‘late’ (green) release zones (produced in ArcMap version 10.5, ESRI, CA).

Two complementary approaches will be used to evaluate the disease impact of *Wolbachia* releases in Medellín and Bello:

i An interrupted time-series analysis utilising routine disease surveillance data collected by the Medellín and Bello Health Secretariats, which aims to compare incidence of dengue, chikungunya and Zika pre- and post- *Wolbachia* release. This analysis will be applied separately to Medellín and Bello.
ii A prospective case control study using a test-negative design, which aims to quantify the reduction in disease incidence among people living within a *Wolbachia*-treated zone compared with an untreated zone that has a similar dengue risk profile at baseline. This study will be conducted in only one quadrant of Medellín ( Figure 1).

### *Wolbachia* deployment

*Wolbachia*-containing adult *Ae. aegypti* mosquitoes and eggs will be deployed sequentially through the study area, starting with the early-release zone of the case-control study (yellow shading in Figure 1; produced in ArcMap version 10.5, ESRI, CA), followed by releases in parts of Medellín and Bello outside the case-control area (grey shading in Figure 1), then lastly in the late-release area of the case-control study (green shading in Figure 1). For the purpose of analysis, release zones will be considered *Wolbachia*-treated from the date of completion of releases. 

An initial period of *Wolbachia* deployment was undertaken in the early-release zone of the case-control area and in parts of Bello between April and December 2017, resulting initially in a high prevalence of *Wolbachia.* However, *Wolbachia* declined after cessation of releases and a subsequent round of deployments was conducted in these areas between mid-2018 and early 2019. It is this deployment period that will be considered for the purpose of the analyses described in this protocol.

### *Wolbachia* monitoring strategy

*Wolbachia* prevalence is monitored through a network of BG-Sentinel adult mosquito traps (BioGents) that are evenly spaced throughout the study area at a density of approximately 16 BG traps per km ^2^. BG traps are serviced weekly, with trapped mosquitoes screened for *Wolbachia* at weekly, fortnightly or monthly intervals throughout the duration of the study, depending on the stage of release and establishment. Trapped mosquitoes will be identified using microscopy. Individual *Ae. aegypti* mosquitoes (male and female) will be tested for *Wolbachia* by quantitative polymerase chain reaction (qPCR) assay. The *Wolbachia* prevalence in screened *Ae. aegypti* will be reported aggregated to the zone (i.e. early- and late-release zone in case-control area) or comuna (for parts of the city outside of the case-control area) level, calculated as the total number of *Ae. aegypti* mosquitoes that tested positive for *Wolbachia* aggregated across all BG traps in the zone/comuna, divided by the total number of *Ae. aegypti* mosquitoes that were screened in that zone/comuna.

### Epidemiological study 1: Interrupted time-series analysis using notifiable disease surveillance data

***Notifiable disease surveillance data.*** In Medellín and Bello, routine public health surveillance for dengue is passive. There are more than 400 public and private health institutions that routinely report clinically-suspected and laboratory-confirmed dengue cases to the Secretary of Health as part of the Epidemiological Surveillance System. Approximately 10% of clinically-suspected dengue cases from hyperendemic or epidemic territories in Colombia are laboratory tested, however this proportion varies and was as high as >60% in 2016 in Medellín. Laboratory evidence suggestive of dengue is usually acquired via detection of anti-dengue IgM antibodies. Laboratory testing for chikungunya and Zika is not routinely performed in Colombia and is only done to demonstrate viral circulation in the area rather than for diagnostic purposes.

For the interrupted time-series analysis, disaggregate (line-listed) data will be requested for notified (clinically-suspected) dengue, chikungunya and Zika cases, and also the subset of dengue cases with IgM ELISA test results, from 2009 to 2025 for both Medellín and Bello. The dataset will include age, sex, address of primary residence, date of illness onset, date of notification, reporting health clinic, disease severity, hospitalisation, death, and, where available, geo-coordinates of the primary residence, type of diagnostic test performed, diagnostic test result, and final diagnostic classification.

***Primary and secondary endpoints.*** The primary endpoint is the incidence of all dengue case notifications to the Epidemiological Surveillance System.

The secondary endpoints are: i) the number of cases who were IgM test positive for dengue; ii) the incidence of severe dengue cases reported to the surveillance system; and iii) the incidence of Zika and chikungunya cases reported to the surveillance system.

***Statistical analysis.*** For the primary endpoint and secondary endpoints above, the impact of *Wolbachia* deployment on disease incidence will be evaluated using an interrupted time series analysis of arbovirus cases reported to the Epidemiological Surveillance System before and after *Wolbachia* establishment. A generalised linear mixed-effects model will be used to model monthly case counts as the outcome variable, with an offset for population size and controlling for seasonality using flexible cubic splines or polynomial functions, to estimate the level change in incidence following the intervention (i.e. completion of *Wolbachia* releases). This analysis will be done separately for Medellín and Bello, 12 months after the completion of releases and each 12 months thereafter until five years post-intervention.

### Epidemiological study 2: Prospective case control study

***Study design.*** The prospective clinic-based case control study uses a test-negative design. The impact of *Wolbachia* deployments on arboviral disease incidence will be assessed by comparing the exposure distribution (probability of living in a *Wolbachia*-treated (early-release) vs. untreated (late-release) area) among virologically-confirmed arboviral disease cases presenting to a network of primary healthcare clinics, against the exposure distribution among patients with febrile illness of non-arboviral aetiology presenting to the same network of clinics in the same temporal window. Arboviral disease cases and arbovirus-negative controls will be sampled concurrently from within the population of patients who reside in the case-control area and present with febrile illness to the study clinic network, with case or control status classified retrospectively based on the results of laboratory diagnostic testing. The distribution of *Wolbachia* exposure in the sampled arbovirus negative controls is assumed to reflect the distribution of *Wolbachia* exposure in the underlying source population that gave rise to cases, as long as a core assumption is met that the relative propensity to seek healthcare for febrile illness at the study clinics in early-versus late-release arms is the same for arboviral disease cases as other febrile illness controls. This should be upheld if cases and controls are clinically indistinguishable until laboratory diagnosis. The concurrent sampling of cases and controls means that the odds of *Wolbachia*-exposure among sampled arboviral disease cases relative to febrile controls (i.e. odds ratio), is an unbiased estimate of the relative incidence of medically-attended arboviral disease in *Wolbachia* early-release versus late-release areas (i.e. relative risk or incidence rate ratio), from which protective efficacy can be estimated directly.

***Study setting.*** The case-control study will be conducted only within a focused study area in northeast Medellín, including six contiguous release zones within four comunas ( Figure 1), with a total population of 580,000 and area 15km ^2^. Among these six release zones, three have been allocated non-randomly as the first zones in Medellín to receive *Wolbachia* deployments (early-release), and three as the last (late-release), such that a parallel two-arm trial is approximated for the period during which *Wolbachia* exposure remains discordant between arms. There are no buffer areas between treatment arms, but natural borders (roads, rivers, non-residential areas) were used to define study arm boundaries as much as possible, to limit the spatial spread of *Wolbachia* from treated areas into untreated areas, and of wild-type mosquitoes into *Wolbachia* treated areas. No attempt will be made to alter the routine dengue prevention and vector control activities conducted by public and private agencies throughout the case-control study area.

***Allocation of the intervention.*** The allocation of the six zones into two arms was done in a way that maximises balance between the arms with respect to measured factors that may be associated with baseline dengue risk, including historical dengue incidence, population characteristics, and geographical area ( Table 1).

**Table 1.** Allocation of the six release zones into ‘early’ and ‘late’ release arms, maximizing balance between the two arms in baseline factors that may predict dengue risk.

		Ratio of baseline characteristics in late/early arms (Ratio of 1 is perfectly balanced)				
‘Early’ Arm	‘Late’ Arm	Aggregate dengue incidence 2013–2016	Population	Area	% population <15 years	Socio-economic status
Aranjuez A, Manrique A, Santa Cruz	Aranjuez B, Manrique B, Popular	1.18	1.02	1.20	1.00	1.11

***Study participants.*** Participants will be invited to participate, by trained research staff, from within the population of patients presenting with undifferentiated fever to a network of primary health care facilities that serve the population who reside in the study area. Participants (or their guardian if <18 years) must provide written informed consent, and meet the following inclusion criteria to be eligible for the study: fever (either self-reported or objectively measured as ≥38 ^o^C), with a date of onset between 1–4 days prior to the day of presentation to the health care facility; aged ≥3 years old; and lived (i.e. slept) in the study area for the 10 days preceding illness onset. Participants will not be eligible for inclusion if localizing features suggestive of a specific diagnosis (e.g. severe diarrhoea, otitis, pneumonia) are identified. An individual presenting to the clinic on repeat occasions for different febrile episodes will be eligible for enrolment during each different episode. However, an individual may only be enrolled once during a single illness episode, which is defined as illness occurring within 4 weeks of a previous febrile episode.

**Figure 2.**
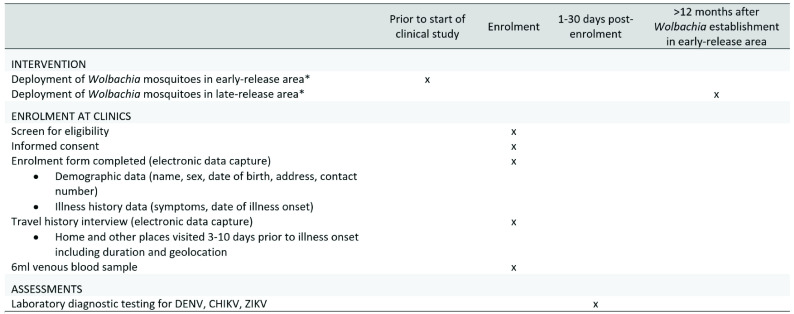
Schedule of enrolment, data collection and assessments (SPIRIT Figure). *Routine dengue prevention and vector control activities will not be altered in treated or untreated areas. DENV: dengue virus; CHIKV: chikungunya virus; ZIKV: Zika virus.

***Data and sample collection.*** A unique identifier will be assigned to each participant at enrolment. Basic demographic details, eligibility against the inclusion criteria, illness onset date, and a retrospective travel history will be recorded in a standardised electronic data collection form. Figure 2 summarises the data and sample to be collected from each participant.

A brief travel history interview will be conducted at enrolment to determine the main places visited by each participant within the 3–10 days prior to illness onset, i.e. the incubation period for dengue. The duration of time spent at home, work or school, and other visited locations during the hours of 5am to 9pm in the 8-day period will be recorded, and the geographic coordinates of those locations derived by geo-locating them on a digital map, with the assistance of the study participant. These data will be used to determine the proportion of time spent in *Wolbachia* treated and untreated areas, for the per-protocol analysis.

**Figure 3.**
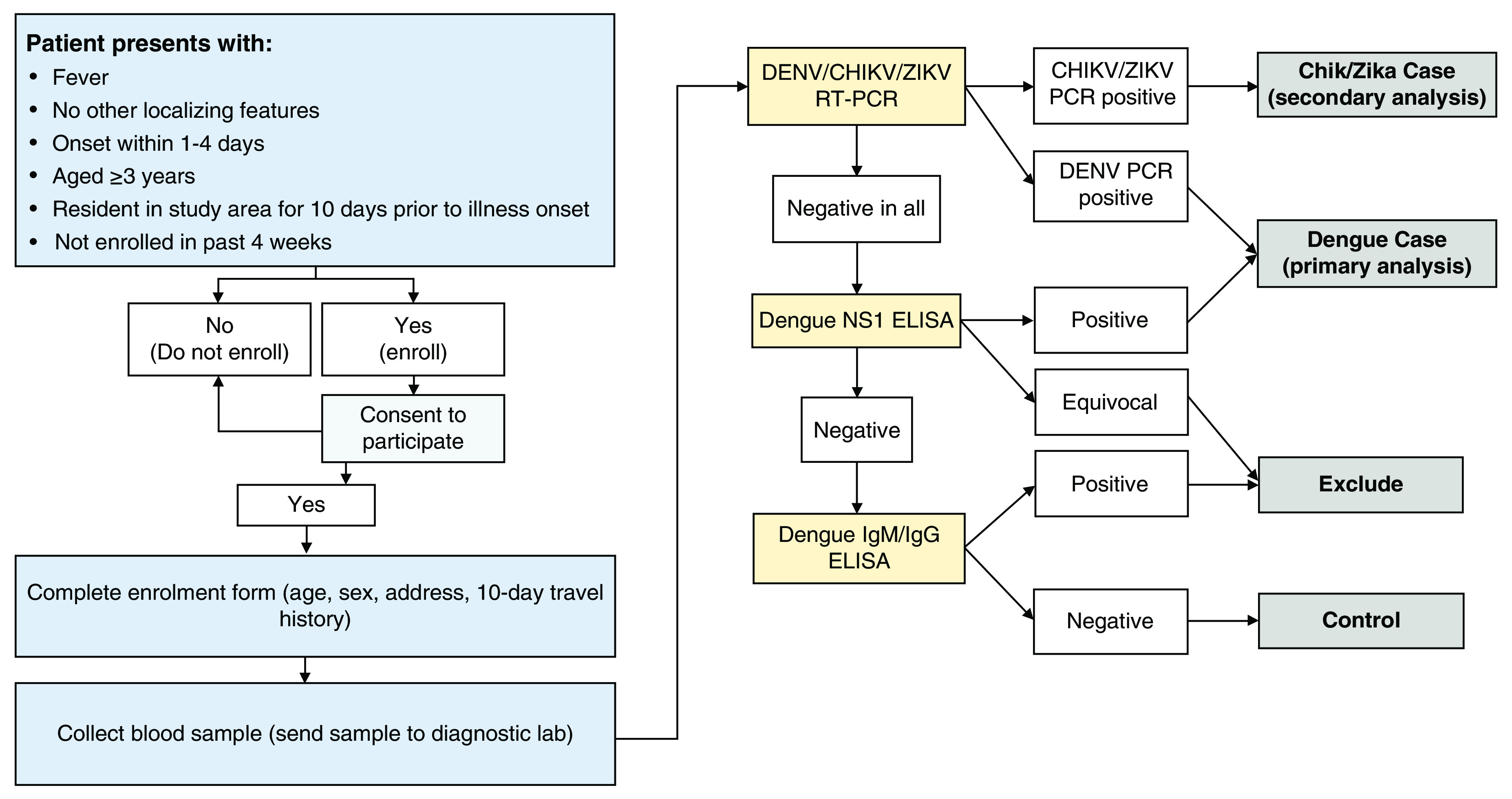
Flowchart of data and sample collection procedures and diagnostic algorithm. Blue boxes indicate participant recruitment and enrolment activities undertaken at clinics. Yellow boxes indicate the laboratory diagnostic testing to be performed at the project laboratory, the results of which (white boxes) will be used to classify participants (grey boxes) as virologically confirmed dengue, Zika or chikungunya cases, arbovirus-negative controls, or excluded due to inability to rule out arbovirus infection according to the algorithm shown. DENV: dengue virus; CHIKV: chikungunya virus; ZIKV: Zika virus; PCR: polymerase chain reaction; NS1: non-structural protein 1; ELISA: enzyme-linked immunosorbent assay; IgM/IgG: immunoglobulin M/G.

A single 6 ml venous blood sample will be collected from all consenting participants on the day of enrolment. Blood samples from all participants will be transferred to the project laboratory on the day of collection and batch-tested within one month to determine case or control status ( Figure 3).

***Laboratory investigations.*** An internally controlled triplex RT-qPCR assay (Bio-Rad) will be used to detect dengue, chikungunya and Zika viruses in serum samples from all enrolled participants. Dengue NS1 ELISA (Dengue Early ELISA, Panbio) will be performed according to the manufacturer’s instructions, on serum samples which have tested negative by DENV RT-qPCR. Dengue IgM and IgG capture ELISA (IgM/IgG Capture ELISA, Panbio) will be performed on serum samples which have tested negative by DENV RT-qPCR and NS1, and would otherwise be classified as controls, to determine whether they have detectable dengue IgM or IgG antibodies indicating potentially acute secondary dengue or another cross-reactive flavivirus infection in order to prevent misclassification. All research diagnostic investigations will be performed by the Programa de Estudio y Control de Enfermedades Tropicales (PECET), at the University of Antioquia, Colombia. 

***Case and control classification.*** Dengue cases are defined as patients with virologically-confirmed DENV infection, meeting the clinical criteria for enrolment and also with a positive result in NS1 ELISA or DENV RT-qPCR. Controls are patients meeting the clinical criteria for enrolment, but with negative test results for DENV RT-qPCR, CHIKV RT-qPCR, ZIKV RT-qPCR, DENV NS1 ELISA, and DENV IgM and IgG ELISA ( Figure 3).

For the secondary endpoints, Zika or chikungunya cases are defined as patients with virologically-confirmed Zika or chikungunya infections, meeting the clinical criteria for enrolment and with a positive result in ZIKV RT-qPCR or CHIKV RT-qPCR, respectively, and controls are defined as above.

**Figure 4.**
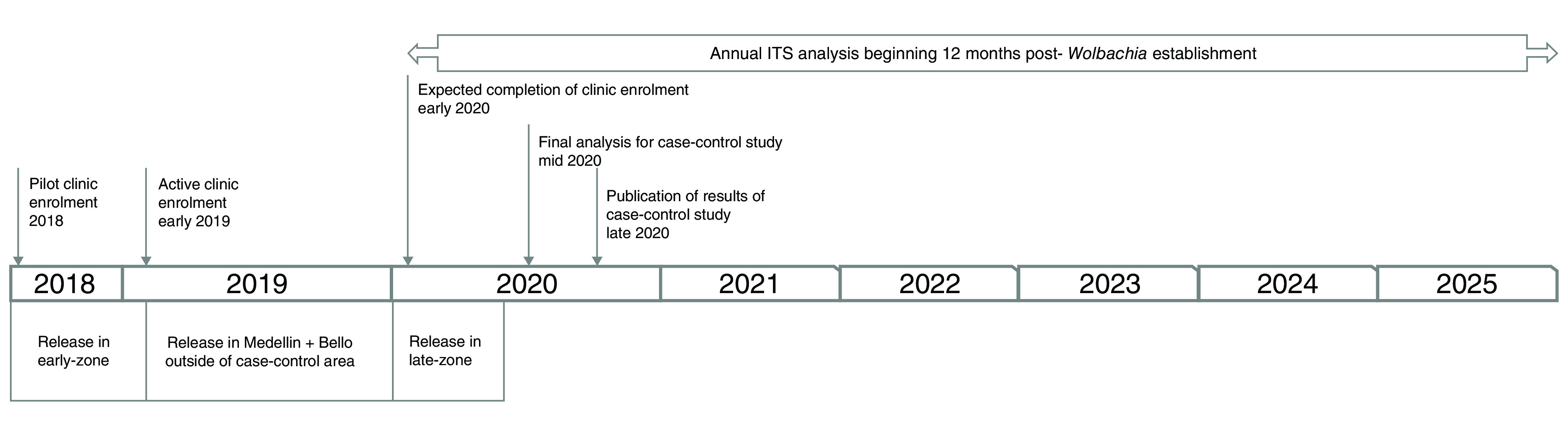
Study timeline. ITS: interrupted time series.

***Expected study duration.*** Pilot clinic-based sampling of febrile patients commenced in November 2017, with enrolment into the intention-to-treat dataset to commence after completion of *Wolbachia* releases in the early-release area. The study will continue to enrol participants until such time as *Wolbachia* deployment commences in the late-release area, which is expected to be at least 12 months after *Wolbachia* establishment in the early-release area, i.e. enrolment can continue even when the target sample size is reached. The study timeline is depicted in Figure 4.

***Power calculations.*** It is estimated that 88 test-positive cases plus four times as many controls will be sufficient to detect a 50% reduction in dengue incidence with 80% power. Thus, we set the target sample size as 100 test-positive dengue cases and expect that by including in the analysis all participants enrolled after *Wolbachia* is established in the early zone, there will be in excess of 400 test-negative controls for 100 test-positive cases. Although we expect the true effect of *Wolbachia* on dengue transmission may be greater than a 50% reduction, the observable reduction in effect is expected to be lower because individuals are likely to spend a substantial proportion of their time outside their release zone of residence. These sample size estimates are based on standard formulae for calculating sample size/power in a case control study. They align with the proposed approach for estimating the intervention effect.

***Statistical analysis.*** The analyses described here will be performed on datasets of cases and controls defined firstly using the primary endpoint of virologically-confirmed dengue cases, and then using the endpoints of virologically-confirmed chikungunya and Zika cases.

The intention-to-treat analysis will consider *Wolbachia* exposure as a binary classification based on residence in the early or late-release area. Residence will be defined as the primary place of residence during the 10 days prior to illness onset. The intervention effect will be estimated from an aggregate odds ratio (for data aggregated across all three *Wolbachia-*release zones within each study arm) comparing the exposure odds (residence in the *Wolbachia* early-release area) among test-positive cases versus test-negative controls. The null hypothesis is that the odds of residence in a *Wolbachia* early-release area is the same among test-positive cases as test-negative controls.

The per-protocol analysis will consider *Wolbachia* exposure as a quantitative index based on measured *Wolbachia* prevalence in local *Ae. aegypti* mosquitoes in the locations visited by the participant during the 10 days prior to illness onset, both within the case control area and in elsewhere in Medellín and Bello. The per-protocol analysis therefore allows for *Wolbachia* exposure to vary in a location over time, and also accounts for human mobility, in terms of the exposure-time that individuals spend outside their area of residence as reported in the travel history interview at enrolment. A weighted ‘ *Wolbachia* exposure index’ (WEI) will be defined for each participant, as follows. The aggregate *Wolbachia* prevalence for each release zone will be calculated each month from all *Ae. aegypti* trapped in that zone. Time spent outside a *Wolbachia* release area will be treated as not *Wolbachia* exposed. The WEI for each participant will then be calculated by multiplying the zone-level *Wolbachia* prevalence (in the month of participant enrolment) at each of the locations visited, by the proportion of time spent at each location, to give a value on a continuous scale from 0 to 1. Cases and controls will be classified by strata of their WEI (e.g. 0-0.2; 0.2-0.4; 0.4-0.6; 0.6-0.8; 0.8-1). This acknowledges that the WEI is not a highly precise measure and serves to reduce error in exposure classification. This analysis can also account for the temporal matching of arboviral disease cases and test-negative controls: risk sets of cases and controls will be defined by frequency matching enrolled confirmed arboviral disease cases to arbovirus-negative controls with illness onset in the same quarter of the year. In the unlikely event that a minimum of four controls cannot be found for a case within the same quarter, the window for matching can be extended until four controls are identified, for that case only. For a time-adjusted analysis, a Cox proportional hazard model will be fitted, which can incorporate the temporal case-control risk sets and participants’ WEI stratum as a categorical variable, using time since completion of *Wolbachia* releases as the time scale.

***Data management.*** Clinical study data will be stored in a custom designed relational database hosted on a secure web-based server. Role-based, tiered access permissions will be used to control access to the clinical database and associated data capture applications. User logs will document the activities of all users. An audit trail will be preserved within the database to capture the history of any changes made to data records after their initial capture.

Data collected from participants in the case-control study will be captured through standardised electronic data capture forms and digital mapping interfaces, deployed as web-based applications on mobile tablets. Laboratory diagnostic results will be captured directly from laboratory assay output and uploaded to a web-based application for storage in the same relational database. Validity controls will be applied at the point of data capture into electronic forms, by predefining value ranges, specifying categorical option lists, and minimizing the use of free text fields. The use of carefully designed electronic forms will facilitate the coding of participant responses at the point of data collection. Quality control in the form of logic and consistency checks will be applied at the point of data capture into an electronic form and at the point of upload into the web-based database. All data relating to the case-control study, including field entomology and epidemiological data, will be retained indefinitely, and for a minimum of five years after study completion, in accordance with International Council for Harmonization on Good Clinical Practice requirements.

***Monitoring of adverse events.*** Any severe adverse events (SAE) associated with collection of blood samples from study participants will be reported to the relevant institutional ethics committees within three days of notification. Standard SAE reporting forms will be used.

***Study governance.*** A steering committee will be assembled to provide operational and strategic advice on planning and operations of the study. A WHO-convened independent evaluation group will review the study within a year of active enrolment as part of a program-wide evaluation of WMP Colombia activities in Medellín and Bello. The independent evaluation group will report its findings to all stakeholders at completion of the review.

***Ethical considerations.*** The study protocol (version 3.0) and the informed consent document have been reviewed and approved by the Institutional Review Boards (IRBs) of the IPS Universitaria of Universidad de Antioquia, Colombia (No. 115, 25 Oct 2017 and No. 127, 12 Oct 2018), and Monash University, Melbourne (ID 11534). Any future protocol amendments will be submitted for review and approval by the same IRBs, prior to implementing protocol changes. The trial protocol was registered on ClinicalTrials.gov (NCT03631719) on 15 ^th^ August 2018.

Confidentiality of participant information will be strictly maintained at all times by the participating investigators, research staff and the sponsoring institution (Universidad de Antioquia) by means of a coded ID number. This confidentiality is extended to cover testing of biological samples in addition to all laboratory specimens, reports, data collection forms and log books, and geolocated records relating to participating subjects. All records that contain names or other personal identifiers, such as informed consent forms, will be stored separately from study records while identified by ID numbers. All local databases will be secured with password-protected access systems. No information concerning the study or the data will be released to any unauthorised third party, without prior written approval of the sponsoring institution. Clinical or personal information will not be released without written permission of the subject, except as necessary for monitoring by an ethical review board or regulatory agencies. Reporting of study results will not be done in any way that permits identification of individual participants, or the location of their homes or other visited locations.

### Current study status

Participant recruitment into the prospective case-control study commenced in early 2019 and is ongoing. *Wolbachia* deployments across the two municipalities are ongoing through to 2020, and collation and analysis of disease surveillance data will continue until 2025.

### Dissemination of study results

Analysis and reporting of the results of the prospective case-control study will occur only at completion of participant enrolment, and subject to the prior approval of the steering committee; there will be no interim analysis or dissemination of results. The interrupted time series analysis will be conducted 12 months after the completion of releases and annually thereafter. Findings from both studies will be submitted for peer review and publication in an appropriate open access journal, together with aggregate supporting data.

## Discussion

Mosquito suppression remains the primary method used to control dengue virus transmission. A recent evaluation found little reliable evidence for the effectiveness of any dengue vector control method, and concluded that standardised studies of higher quality must be prioritised ^9^. *Aedes aegypti* mosquitoes are the primary vectors of dengue, chikungunya and Zika viruses, therefore evidence-based interventions targeting this species have the potential to reduce multiple arboviral diseases where they co-circulate. The study described here will evaluate the impact of *Wolbachia*-infected mosquitoes on dengue and other arboviral diseases in Bello and Medellín municipalities, Colombia, using a combination of routinely collected disease surveillance data throughout the municipalities, and a prospective clinic-based test-negative study focused in one area of Medellín.

The test-negative design, a variant on the case-control design, which has been widely applied in non-randomised influenza vaccine effectiveness studies, uses outcome-based concurrent sampling of dengue cases and non-dengue controls to measure the efficacy endpoint ^23– 26^. Effect estimates (odds ratios) from a test-negative design are equivalent to direct estimates of relative risk in the source population, under the assumption that the distribution of test-negative illness is not associated with the intervention, and that test-negative controls are allowed to include participants who may be classified as dengue cases at other times during the study period.

The interrupted time-series design is commonly used to evaluate the impact of public health interventions introduced at a population level and targeting population-level health outcomes ^27– 29^. A series of repeat observations over time is analysed to establish a baseline trend which is assumed to be ‘interrupted’ by the introduction of an intervention. The subsequent post-intervention trend is compared to the counterfactual that would be expected in the absence of the intervention based on the baseline trend. A quantitative estimate of the intervention effect is derived from a segmented linear regression model of the outcome of interest (e.g. dengue case count or incidence) as a function of time, in which the intervention status is captured by a binary variable coded 0 for pre-intervention time points and 1 for post-intervention time points ^30, 31^. Seasonality and other secular trends and time-varying confounders can also be controlled for by the inclusion of additional model parameters. A limitation of using notifiable disease surveillance data is the imperfect specificity of the clinical case definition used for notifications, meaning an unknown and time-varying proportion of notified cases are not true dengue infections. Inconsistent reporting practices, outbreaks of non-dengue febrile diseases, or other factors may induce secular trends in the surveillance data that are independent of, but contemporaneous with, the *Wolbachia* intervention and thus may influence our ability to estimate the true intervention effect from time series data. A subset of the notified dengue cases in Colombia have supportive laboratory diagnostic results, but these have several limitations: i) laboratory testing can be infrequent, particularly during outbreaks, ii) the cross-reactivity of IgM serology between dengue and Zika limits the utility of serological data where Zika co-circulates, and iii) no virological confirmation (PCR or NS1 antigen detection) of cases is performed. We therefore base our primary analysis on all notified dengue cases (suspected and dengue IgM test positive). The benefits of using these routinely collected surveillance data include the availability of a long time series, reduced costs for data collection and timely acquisition of data.

The pragmatic approach described here to evaluate disease impact arose from the imperative from funders and local stakeholders to achieve rapid scale-up of *Wolbachia* deployments and to retain flexibility in the release sequence and methods, in the context of the declaration of the Zika public health emergency at the time of project conception and funding ^8^. These imperatives precluded implementation of the proposed test-negative design study across all of Medellín/Bello, given the time required to obtain approvals, establish clinical enrolment processes, train staff, and then maintain an untreated comparison area for the duration of clinical enrolment. Randomised allocation of the early and late *Wolbachia* release areas in the focused case-control study was also not feasible, given the small number of zones within the case-control area. In general, there is greater potential for selection bias in non-randomised studies than in randomised studies, which, in the context of the current study, may present as a differential distribution of non-arboviral febrile illness (i.e. test-negative controls) by study arm due to chance imbalance in the care-seeking populations between study arms or differential propensity for care-seeking among those in areas where *Wolbachia* is released compared with untreated areas. As long as a core assumption of the test-negative design is met that the relative propensity to seek healthcare for undifferentiated febrile illness at a study clinic, in early vs late zones, is the same for dengue cases as for other febrile illness controls, then an imbalance in participant enrolment between early and late zones should not in itself bias the results.

A staged approach to city-wide deployments following a ‘stepped-wedge’ design was also considered in planning the disease impact assessment for Medellín, however this carried a requirement to define a deployment sequence that maintained balance between ‘already treated’ and ‘not yet treated’ areas in factors associated with baseline dengue risk, to avoid selection bias. This had resource implications for the field entomology and community engagement teams’ activities that were inconsistent with the necessarily pragmatic approach to deployment in Medellín and Bello needed to achieve large-scale coverage within a short time frame.

The non-blinded deployment of the intervention means community members may alter their care seeking or vector control behaviour due to the belief that they are protected from dengue by *Wolbachia*. Blinding was determined to be cost prohibitive in this study as it would have doubled the resources and time required to conduct field releases of mosquitoes. Under the test-negative design, test-positive cases and test-negative controls are drawn from the same population of patients presenting to health clinics with febrile illness, who are clinically indistinguishable at the time of enrolment. Thus, the threat of bias due to non-blinding in the test-negative study is considered minimal as long as any modified behaviour applies equally to test-positive cases and test-negative controls. Routine vector control by health authorities were not altered as part of the study, and it is possible that some public health activities or change in community behaviour occurred which impacts arboviral disease incidence independently of *Wolbachia*, leading to an under- or over-estimation of the *Wolbachia* intervention effect in our study.

Human mobility is a potential confounding factor in the estimation of *Wolbachia*’s impact on arboviral disease. Individuals in whom the efficacy endpoint is measured may spend a proportion of their time outside their allocated study area (i.e. their area of primary residence) resulting in at least some exposure misclassification. There are no buffer zones separating the early-release and late-release areas of the case control study, nor the other areas of Medellín and Bello where staged deployments will occur, and *Wolbachia* contamination from one area to another is possible but also measurable. The combined effect of human mobility and *Wolbachia* contamination is that the exposure status of the populations in the nominally ‘treated’ and ‘untreated’ areas become more similar to each other, thereby diluting any estimated intervention effect towards the null. By powering the case-control study to detect a relatively conservative effect size of 50%, we have allowed for some of this effect dilution while targeting a reduction in dengue incidence of public health significance. The case-control study per-protocol analysis, where recent travel history is documented and a quantitative *Wolbachia* exposure status is calculated for each participant, will account for both for the time spent in areas away from home and the local measure of *Wolbachia* prevalence in visited areas.

Inter-annual fluctuations in dengue transmission mean that the case-control study might fall in a period of lower incidence just by chance. Nevertheless, the target sample size of 100 dengue test-positive cases is seen as feasible even in the event of a low transmission year. The interrupted time-series analysis, which spans a longer period than the case-control study, is expected to have at least three to five years of post- *Wolbachia* release data, and thus should be more tolerant to periods of low dengue transmission.

The generalisability of the current study’s findings to other dengue endemic settings will likely be influenced by setting-specific factors such as the local entomological context, which influences *Wolbachia* introgression. Generalisability may also depend on the local distribution of circulating DENV serotypes and the intensity of virus transmission, which might influence the observed impact of *Wolbachia* on arboviral disease incidence. A cost-effectiveness analysis is underway in Indonesia, and the findings are expected to help inform cost optimisation for different deployment scenarios, target settings, and scale-up methods that can be applied to other countries. If the results of the current study do demonstrate a reduction in arboviral disease incidence associated with *Wolbachia* deployment in Medellín and Bello, a key next step would be to scale this intervention into wider public health implementation in Colombia and elsewhere in the Latin American region, using epidemiological, ecological and cost-effectiveness data to inform an optimal strategy for scaled deployment.

### Ethics approval and consent to participate

This study has been approved by the Institutional Review Boards of the IPS Universitaria of Universidad de Antioquia, Colombia, and Monash University, Melbourne. Written informed consent will be obtained from all participants, or their guardian where the participant is a minor (<18 years), prior to enrolment in the clinic-based case-control study. The interrupted time-series analysis uses pre-existing non-identifiable disease surveillance data, which does not require individuals’ consent.

## Data availability

No data is associated with this article.
