# Supplementary material for: The impact of city-wide deployment of Wolbachia-carrying mosquitoes on arboviral disease incidence in Medellín and Bello, Colombia: study protocol for an interrupted time-series analysis and a test-negative design study
Source: F1000Res. 2020 May 21;8:1327. Originally published 2019 Aug 1. [Version 2] doi: 10.12688/f1000research.19858.2 (PMC8637243; doi:10.12688/f1000research.19858.2)
Supplement: Peer review history

# Peer review: Reviewer response for version 2

**DOI:** 10.5256/f1000research.26341.r100109

**Published:** 2021-12-01

**Authors:** Amy Morrison

**Affiliations:** 1 Department of Pathology, Microbiology and Immunology, School of Veterinary Medicine, University of California, Davis (UC Davis), Davis, CA, USA

The revised study protocol “The impact of city-wide deployment of *Wolbachia*-carrying mosquitoes on arboviral disease incidence in Medellín and Bello, Colombia: study protocol for an interrupted time-series analysis and a test-negative design study” describes a pragmatic study designed to evaluate the impact of releases of *Aedes aegypti* stably transfected with the *Wolbachia* bacterium on the incidence of three *Aedes* transmitted viral diseases (ATVDs - dengue [DENV], chikungunya [CHIKV], and Zika [ZIKV]). There is and has been a critical need for evidence of Public Health Impact for vector-borne diseases for both traditional and novel vector control strategies. The *Wolbachia* releases described herein fall in the later category and the World Mosquito Program (WMP) continues to publish studies that indicate that this strategy has significant promise for controlling ATVDs in certain locations and ecologies. Although the authors provide a convincing argument for why they were unable to conduct a more rigorous randomized control trial design in this site. Because a cRCT is underway in Brazil and randomized control trial that also used the same test-negative design in Indonesia are providing high-level evidence that this vector control strategy is effective, I am supportive of developing alternative kinds of evidence which this study does (Utarini *et al.* (2021 ^1^)). I can personally attest to the huge difficulties associated with carrying out and properly powering a cRCT for ATVDs, so the group's effort to develop more a pragmatic approach is a valuable contribution to the field. I would encourage the authors to suggest their study design for ongoing evaluations of traditional and novel vector control being deployed by government programs. When taking a pragmatic approach such as this using multiple approaches is far more credible than a single approach (test-negative case control study and  interrupted time series analyses here). A third source of evidence that would have been even more convincing would have been a pediatric cohort in each of the 4 zones to measure seroconversion to ATVDs, preferably in a population who was seronegative at baseline. I would argue that it might provide a faster less bias estimate of the impact of *Wolbachia* releases on transmission between the two allocation areas. To be clear, the approach and study design is reasonable, especially if the 4 zones are comparable, which the authors indicate they are. I would however ask that the authors address the following critical questions.

 Are other vector control activities being conducted during the pre- and post- release periods? How do these activities potentially impact the interpretation of both the interrupted time series and test-negative test? I think it is important to provide some evidence that these government measures, effective or not, are applied equally across the 4 zones used for the test-negative study and that any impact observed in the interrupted time series analysis is not due to ongoing activities especially if government programs have improved since releases began. My suspicions are these arguments can be made but controlling for or at least considering these activities should be addressed in the article.

 The article/protocol focuses on the human component of the study which is appropriate, however, more details on the release strategy and approach would provide a more complete picture. The most important issue here is *Wolbachia* prevalence.  I think this document would benefit from a clear description of the minimum *Wolbachia* prevalence required by the design and the range (expected variation) of prevalence expected.  Although an earlier release in which *Wolbachia* did not become established is mentioned, the implications of this are important.  What happens if *Wolbachia* does not become established in any of the negative test zones?

 I have some concerns about the WEI. From a theoretical perspective this looks great, but inclusion (as an appendix) of the human mobility survey would be helpful.  In my experience it takes considerable effort to confirm all secondary locations and to estimated time in the two allocation arms will be quite variable over the 10-day period. I commend the authors for attempting to account for the fact that participants will not always be in a "protected" area and can be exposed to ATVDs outside of the zone of their primary residence. More details on this questionnaire would be appreciated.  Additionally, how missing, or incomplete data is handled here would be useful. You are really asking what proportion of time is spent in study zone versus outside it, and if outside means an untreated area or not..

 Although I do not think I was able to access the full written responses to the previous reviewers, the current version does address most of the issues raised. It also provides a discussion of many of the design limitations, of which there are many. I say that not as a criticism, because it is widely accepted that surveillance programs are severely flawed, presenting a variety of challenges to proper evaluation of disease endpoints. If the results from the proposed study are viewed carefully, and the final analyses clearly described without bias, this study will provide important data on the efficacy of *Wolbachia* releases. The statistical analysis presented in this article, has a lot of built in flexibility, which I'm not opposed to, however, this does open the authors up to criticism for what might look like data massaging or selecting the approach that will look most favorable. The details of the per protocol analysis will need to disclosed in detail when the studies are published.

 Below are some specific comments and observations from the text:

- **Background:**
  - Paragraph 4: The sentence, “The mean annual incidence of notified dengue cases in the seven years 2010–2016 prior to the start ofscaled *Wolbachia* deployments was 298 per 100,000 population in Medellin (range 38 – 771 per 100,000) and 188 per 100,000 population in Bello (range 36 – 446 per 100,000)”, is duplicated.  Eliminate the first which also includes a typo. 
  - Table 1. Could information also be stratified y allocation. This table could be structured differently to provide both the aggregated, allocation-specific data, and the 4 zones selected for the study.
- **Laboratory Investigations:**
  - I would be more comfortable if paired acute and convalescent samples could have been collected here.  If the inclusion criteria are applied consistently across the same zones and overtime, I believe you are detecting a real signal here but there is the potential for misclassification of true dengue cases as negative controls. The exclusion of the equivocal and dengue IgM/IgG ELISA positive will reduce this possibility but reporting how many of these individuals are excluded should be done when reporting the final study results.
  - I would have liked to see a contingency for situations with confirmed CHIKV/ZIKV outbreaks. That is define criteria, when added CHIKV/ZIKV testing would be done.  Again, your approach is consistent and still identify signal differences between the two allocation arms, but with ZIKV the time window to detect acute infections by PCR can be short. I would only suggest this during periods of high and known transmission which I assume have not been detected during the follow up period yet.
- **Expected study duration:** Will deployment in the late release areas be delayed if the target sample size is not reached?
- **Correct typo below:** “The study will continue to enrol participants until such time as *Wolbachia* deployment commences in the late-release area”.
- **Statistical Analysis Plan:** Calculation of the WEI: From a theoretical perspective this looks great, but inclusion of the human mobility survey would be useful.  Also, in my experience it takes considerable effort to confirm all secondary locations and estimating time in the two allocation arms will be quite variable over the 10-day period.
- **Monitoring of adverse events:** Do you expect any Severe Adverse Events? What are they and how would you monitor for them?  Will you have any contact with participants after they provide the blood sample. Do you provide results. Clearly this is standard language, but it would be more appropriate the indicate what adverse events are expected and what would constitute and Severe Adverse Event in the context of the study.  For example, would a participant who develops severe dengue, becomes hospitalized, and needs ICU care be reported in this context?
- **Ethical considerations:** Is there any national or government approvals required and if so, what? This study is clinical research but a clinical trial.

Is the study design appropriate for the research question?

Yes

Is the rationale for, and objectives of, the study clearly described?

Yes

Are sufficient details of the methods provided to allow replication by others?

Partly

Are the datasets clearly presented in a useable and accessible format?

Not applicable

Reviewer Expertise:

I have conducted prospective epidemiological studies on dengue and its vector Aedes aegypti in the city of Iquitos, Peru since 1998.  I have conducted two cRCTs with epidemiological endpoints for insecticide treated curtains and spatial repellents against ATVDs as well as other vector control trials

I confirm that I have read this submission and believe that I have an appropriate level of expertise to confirm that it is of an acceptable scientific standard.

## References

[1] : Efficacy of Wolbachia-Infected Mosquito Deployments for the Control of Dengue. *New England Journal of Medicine* .2021;384(23) : 10.1056/NEJMoa2030243 2177-2186 10.1056/NEJMoa2030243 PMC810365534107180

# Peer review: Reviewer response for version 2

**DOI:** 10.5256/f1000research.26341.r63750

**Published:** 2021-06-11

**Authors:** Stephen Waterman

**Affiliations:** 1 CDC Dengue Branch, Centers for Disease Control and Prevention (CDC), San Juan, Puerto Rico

This manuscript describing the protocol for a intervention trial of *Wolbachia* replacement in *Aedes aegypti* populations with the wMel Wolbachia strain to prevent dengue, Zika and chikungunya in the complicated densely populated urban environments of Rio de Janeiro and Niteroi, Brazil, has been revised somewhat in response to previous open reviewers.    In response to both reviewers, the authors explain the pragmatic reasoning behind not conducting the study as a cluster randomized trial.  In addition to community pressure to deploy the intervention quickly and in wide areas as a result of the Zika outbreak, the authors indicate that the necessary community engagement to set up clusters was not feasible given the resources available, and that the smaller geographic units are more susceptible to contamination.  In response to reviewer 1 the authors clarify the *Wolbachia *strain used and state that the wMel strain has persisted at high levels for over two years in Indonesia and as many 8 years in northern Australia. 

 Each city has four intervention release zones (ranging from 9 to 51 square kms and populations densities from 1,400-18,000 persons/square km) and a large non-release control area.  Adult mosquito releases took place between late 2015 and the end of 2019 and lasted for at least 16 weeks in each release zone. *Wolbachia *introgression frequency monitoring is done with BG traps and PCR testing of a sample of the mosquitoes collected. The study design main analysis methodology is controlled interrupted time series which makes use of routinely collected public health disease surveillance data and requires that consistent historical data is available supporting the comparability of the intervention and control areas. The analysis compares the incidence of suspect and confirmed dengue cases before and after the intervention in the treatment and control zones.  Additional details on the regression analysis have been provided. Power estimates from simulations based on binomial distribution of ten year historical data suggest that the study has 80% to detect a 50% reduction after three years, and a 60% reduction after two years. 

 This study is important to assess the impact of *Wolbachia *replacement in a highly *Aedes aegypti* transmitted disease endemic urban area in Latin America, as the other studies conducted by the World Mosquito Program have been done in Asia or in non-endemic Australia.  Since the study is not randomized, including as much detail as possible in the description of the methods and the results in eventual publications will be important to assess the strengths and weaknesses of the study. 

**Comments:**

- Additional details regarding the *Wolbachia *mosquito releases and trapping results would be of interest.  Why were releases done as adult mosquito releases and not eggs as in the Yogyakarta Indonesia RCT trial? (Anders *et al.* (2020 ^1^))  Were releases in all release zones begun simultaneously or staggered?   Were the BG trap adult female *Aedes aegypti* mosquito counts/densities comparable in the different zones and control area?  Will the study design enable assessment of the impact of *Wolbachia *releases on *Aedes* species abundance?
- Large dengue outbreaks had not occurred prior to the intervention in the study cities since 2013.  Is there available seroprevalence data for dengue and Zika in the intervention zones and the control area prior to the wMel intervention?  Do we know if pre-intervention reported Zika incidence in 2016 was comparable in the intervention and control areas? 
- The analysis plan is to report results two years after completion of releases. If the releases were staggered will the initial publication include data from all the intervention zones? 
- Are the secondary endpoint analyses of severe dengue cases and fatalities intended to strengthen the efficacy evidence with data on confirmed diagnoses, to help address the cost effectiveness of the intervention, or to address a question whether *Wolbachia *reduces the severity of disease when transmission does occur?   
- The protocol states that the study will continue through 2023 and analyses will be done each year for 5 years.  Since the releases finished at the end of 2019, will an analysis of the sustained *Wolbachia *introgression and cumulative epidemiologic impact after 5 years be possible?   

**Minor comments:**

- The statement in last paragraph of page 9 that we have no evidence of dengue, Zika or chikungunya transmission where high levels of *Wolbachia *have been established is somewhat misleading in that the authors are referring to northern Australia where *Aedes aegypti* transmitted diseases are not endemic and the force of infection is considerably lower than Asia and Latin America. 
- The comment on page 9, next to last paragraph with regard to the licensed CYD-TDV vaccine being rendered less feasible in high burden areas because of the need for pre-vaccination screening actually better applies to low burden areas.  WHO included in its 2018 vaccine recommendations (WHO (2018 ^2^)) that areas with dengue seroprevalence of 80% or greater in 9 years might consider forgoing pre-vaccination screening.  In low burden regions the risk of vaccinating false serologic positives increases and the cost effectiveness decreases.  The authors may want to clarify whether they think *Wolbachia *replacement could make dengue vaccines unnecessary.   

Is the study design appropriate for the research question?

Yes

Is the rationale for, and objectives of, the study clearly described?

Yes

Are sufficient details of the methods provided to allow replication by others?

Partly

Are the datasets clearly presented in a useable and accessible format?

Not applicable

Reviewer Expertise:

Epidemiology, dengue and arbovirology, tropical medicine

I confirm that I have read this submission and believe that I have an appropriate level of expertise to confirm that it is of an acceptable scientific standard.

## References

[1] : Update to the AWED (Applying Wolbachia to Eliminate Dengue) trial study protocol: a cluster randomised controlled trial in Yogyakarta, Indonesia. *Trials* .2020;21(1) : 10.1186/s13063-020-04367-2 429 10.1186/s13063-020-04367-2 32450914PMC7249400

[2] : Weekly epidemiological record, No 36, 7 September.2018; Reference source

# Peer review: Reviewer response for version 1

**DOI:** 10.5256/f1000research.21786.r57177

**Published:** 2019-12-16

**Authors:** Penelope Hancock

**Affiliations:** 1 Big Data Institute, University of Oxford, Oxford, UK

The paper describes protocols for assessing the efficacy of *Wolbachia* releases on reducing dengue incidence based on data from release trials conducted in municipalities in Columbia. The *Wolbachia* has been released into 6 adjacent zones covering a sub-area, where 3 of the zones have early releases and the other 3 have later releases – the zones which receive late releases are regarded as non-intervention arms for the purposes of the trial analysis.

 It is always difficult to measure the efficacy of vector control interventions using randomized trials because of community level effects such as herd immunity, so that the intervention has impacts on both treatments and controls, and the estimated effect of the intervention is diluted – an effect known as contamination. In the case of *Wolbachia* releases, the effect is particularly difficult to quantify because the *Wolbachia* may spread to mosquito populations in the control arms, or *Wolbachia* may only have an intermediate (and variable) prevalence in the intervention arms. The trial described in this paper has additional complications which the authors describe in the manuscript, including the non-random allocation of intervention clusters and the small size of the study area with intervention arms adjacent to control arms and no buffer between them. The authors explain that these limitations mean that a pragmatic approach to analysis of *Wolbachia* efficacy is required.

 I think the paper would benefit from a more rigorous quantitative protocol for how intervention efficacy can be quantified in the presence of contamination (see Silkey *et al*. 2016, Trials ^1^). The power calculation that the authors provide doesn’t explore impacts of contamination, or impacts of incomplete spread of *Wolbachia* in the intervention arm. In addition the issue of herd immunity should be pointed out in the paper. It seems that given the configuration of the release zones, an effective *Wolbachia* intervention could greatly interrupt dengue transmission in the non-intervention (later release zones) as well, thus making efficacy very difficult to evaluate.

 I find the *Wolbachia* Exposure Index proposed for individual participants (to account for their movements outside/into release zones) to be problematic. *Aedes aegypti* have a very heterogeneous local distribution, and I don’t think variations in exposure can be calculated using travel histories – these estimates are likely to be misleading at best.

 Regarding the interrupted time series approach, I think a more detailed and formal mathematical description of the method is needed, detailing the sampling distribution of the infection data with respect to the location of *Wolbachia* interventions, and the statistical model for handling spatiotemporal autocorrelation. It does not seem straightforward to distinguish the effect of *Wolbachia* from non-related fluctuations in disease incidence caused by environmental factors. More explanation of how this will be achieved is required.

 Please put a scale on the map in Figure 1.

Is the study design appropriate for the research question?

Partly

Is the rationale for, and objectives of, the study clearly described?

Yes

Are sufficient details of the methods provided to allow replication by others?

No

Are the datasets clearly presented in a useable and accessible format?

Not applicable

Reviewer Expertise:

Spatial modelling and statistics, population dynamics, Wolbachia.

I confirm that I have read this submission and believe that I have an appropriate level of expertise to confirm that it is of an acceptable scientific standard, however I have significant reservations, as outlined above.

## References

[1] : Design of trials for interrupting the transmission of endemic pathogens. *Trials* .2016;17(1) : 10.1186/s13063-016-1378-1 10.1186/s13063-016-1378-1 27266269PMC4895826

# Response

**Published:** 2020-05-13

**Authors:** Katherine ANDERS

**Affiliations:** Monash University, Australia

*I think the paper would benefit from a more rigorous quantitative protocol for how intervention efficacy can be quantified in the presence of contamination (see Silkey et al. 2016, Trials).*

Authors’ response:

 Contamination will be addressed in the analysis by incorporating area-level measured *Wolbachia *frequencies in the model, rather than presuming an area’s exposure status based on whether *Wolbachia* releases had occurred in that area. For the interrupted time-series analysis of dengue cases notified to the Medellin and Bello health surveillance system, additional analyses will now explore the effect of *Wolbachia *on the incidence of notified dengue using *Wolbachia *frequency either as a continuous covariate or categorised into quintiles of exposure. In the per-protocol analysis of the case-control study, each participant will be assigned a *Wolbachia* Exposure Index calculated using zone-level *Wolbachia* frequency and time spent in that release zone. These methods thus additionally take into account potential incomplete spread of *Wolbachia* in release areas as well as contamination into areas where *Wolbachia* releases have not yet occurred.

 The method proposed by Silkey *et al* to quantify the magnitude of the contamination effect – i.e. comparing disease incidence among naïve (non-intervened) groups living close to an intervention area to those in naïve groups living remote from the intervention - cannot account for the possibility that *Wolbachia* fails to establish in a communa where releases have occurred and would instead assume these communas to be *Wolbachia*-treated.

*The power calculation that the authors provide doesn’t explore impacts of contamination, or impacts of incomplete spread of Wolbachia in the intervention arm.*

Authors’ response:

 Contamination and/or incomplete spread of *Wolbachia *is expected to dilute any intervention effect by making the intervention and untreated areas more similar. Our sample size is powered to detect the conservative effect size of 50%, though we expect the true reduction in dengue incidence to be much greater than 50% based on preliminary findings from other WMP project sites which demonstrate reductions ranging from 75% to 97% at 12 months or more post-intervention. Our existing sample size therefore allows for a dilution of the true intervention effect by contamination, incomplete *Wolbachia *establishment in intervention areas, and/or human mobility.

*In addition the issue of herd immunity should be pointed out in the paper. It seems that given the configuration of the release zones, an effective Wolbachia intervention could greatly interrupt dengue transmission in the non-intervention (later release zones) as well, thus making efficacy very difficult to evaluate.*

Authors’ response:

 Dengue transmission is known to be quite focal (<500m) in urban areas (Salje et al PNAS 2012; Morrison et al PloS NTD 2010) so it is unlikely, although possible, that interruption of transmission in the early release zone would have spillover effects into the adjacent late release areas, independent of the potential movement of the *Wolbachia *mosquitoes themselves. A brief discussion has been added to the paper (see Discussion, paragraph 7).

*I find the Wolbachia Exposure Index proposed for individual participants (to account for their movements outside/into release zones) to be problematic. Aedes aegypti have a very heterogeneous local distribution, and I don’t think variations in exposure can be calculated using travel histories – these estimates are likely to be misleading at best.*

Authors’ response:

 The *Wolbachia* Exposure Index aims to capture not only human mobility in and out of release zones, but also heterogeneity in *Wolbachia* establishment among the early-release zones. The WEI will be calculated using the zone-specific aggregated *Wolbachia* prevalence in the month of participant enrolment, which recognises that *Wolbachia* establishment in intervention areas may be imperfect, that some *Wolbachia* spread into the control area may occur, and that *Wolbachia* prevalence varies across time. We acknowledge that there will be heterogeneity in *Wolbachia* frequency even within each zone (and also in *Ae. aegypti* distribution as the reviewer notes), however, no accurate interpolation at a geographical scale smaller than communa level is possible.

 We have added an additional per-protocol analysis in which the WEI is calculated using only the zone-level *Wolbachia* prevalence in the participant’s zone of residence (in the month of participant enrolment), ignoring the participant’s recent travel history. This recognises that dengue exposure risk may be higher at home versus other locations, rather than assuming an even distribution of exposure risk across daytime hours and locations visited (see paragraph 4 of Methods – Statistical analysis).

*Regarding the interrupted time series approach, I think a more detailed and formal mathematical description of the method is needed, detailing the sampling distribution of the infection data with respect to the location of Wolbachia interventions, and the statistical model for handling spatiotemporal autocorrelation. It does not seem straightforward to distinguish the effect of Wolbachia from non-related fluctuations in disease incidence caused by environmental factors. More explanation of how this will be achieved is required.*

Authors’ response:

 We previously planned on using a mixed-effects negative binomial model to assess the impact of the *Wolbachia* intervention on dengue incidence, modelling between-communa variability as a random effect by including a random intercept at the communa level and allowing for a random-slope on the intervention. However, given that modelling the random effects themselves are not of particular interest in this analysis, it was decided that a generalised estimating equation approach would be more efficient as it most directly targets the group-level parameter of the intervention effect and requires fewer modelling assumptions than a mixed-effects model. The outcome distribution is assumed to be negative binomial to allow for overdispersion. Monthly case counts will be modelled as the outcome variable, with an offset for population size and controlling for seasonality using flexible cubic splines. Robust standard errors will be used to account for clustering of cases by communa. An autoregressive correlation structure will be specified to account for temporal autocorrelation. A mixed-effects model will then be used to check for potential small sample issues in inference estimation, given the modest number of clusters in Medellin (18 communas) and Bello (11 communas) and the large number of observations per cluster.

 We acknowledge that there may be other environmental factors contributing to natural fluctuations in dengue incidence which we are unable to explicitly account for in the model. The availability of historical data (range 5-8 years) and the staged nature of the intervention means the statistical model includes between-communa (within-period) comparisons as well as before-and-after (within-communa) comparisons, which is expected to control for at least some of these natural fluctuations that are unrelated to the *Wolbachia* intervention.    

*Please put a scale on the map in Figure 1.*

Authors’ response:

 A scale has been added to the map.

# Peer review: Reviewer response for version 1

**DOI:** 10.5256/f1000research.21786.r51965

**Published:** 2019-08-28

**Authors:** Stephen Waterman, Roberto Barrera

**Affiliations:** 1 CDC Dengue Branch, Centers for Disease Control and Prevention (CDC), San Juan, Puerto Rico; 2 CDC Dengue Branch, Centers for Disease Control and Prevention (CDC), San Juan, Puerto Rico

This paper describes the protocol for a clinical trial releasing *Wolbachia* bacterium infected male and female *Aedes aegypti* mosquitoes in Medellín and Bello, Colombia, with the primary endpoint objective of demonstrating reduction of human dengue infections in the population living in the treated areas. *Wolbachia* infected female release over time results in replacement of the uninfected *Aedes aegypti* population with *Wolbachia* infected mosquitoes that have reduced ability to transmit arboviruses. The study design has two components, an interrupted time series (ITS) approach using passive arboviral disease surveillance data from Bello and a large area of Medellín, and a prospective case-control in a smaller area of northeast Medellin using arbovirus laboratory test negative acute febrile illness controls in 6 early release and late release zones.  The studies begin approximately one year after mosquito releases (in 2018 for the early release zone and 2019 for the releases outside the case-control area) to allow for mosquito population replacement. 

 This study employs a novel *Aedes aegypti* vector control approach to address the major and growing tropical public health problem of *Aedes* transmitted arboviruses including dengue, Zika, and chikungunya viruses.  The authors point out that such studies are badly needed as conventional *Aedes aegypti* vector control approaches have failed or been shown to be non-sustainable, and the only currently licensed dengue vaccine is partially effective and requires a laboratory test prior to immunization.   

 This study protocol builds on laboratory and fieldwork in Australia and elsewhere, and is well designed and well written with considerable detail (design, statistical power and analysis, laboratory testing, data management, etc) to address the question of human arboviral disease impact of *Wolbachia* replacement in a highly endemic tropical urban setting in Latin America.   The protocol is intentionally “pragmatic” to roll out the intervention at large scale under operational conditions in a timely and cost effective manner acceptable to the study community. The authors thus effectively answer potential criticisms of the study design not being a cluster randomized clinical trial as recommended by the WHO Vector Control Advisory Group. The case control study design also importantly accounts for the extent of replacement with *Wolbachia* infected mosquitoes in the treated area and human mobility out of the treatment area with a calculated *Wolbachia* exposure index calculated for each acute febrile illness patient. 

 The test negative case control study design obviates the need for expensive clinical cohort follow up to monitor disease incidence and treated and untreated areas. The authors correctly point out that the risk estimate from the test negative case control approach is likely to be unbiased and can be used to calculate efficacy confidence limits. The ITS approach is a less rigorous experimental design in that the vast majority of passively reported cases are not virologically confirmed, and surveillance may not be always be done in a consistent manner, but is another practical approach using available data from the Ministry of Health that allows for monitoring of reductions in arboviral case reports over a long time period (5 years) after the *Wolbachia* replacement treatment during which arbovirus outbreak cycles would be expected to occur.  

 The discussion appropriately comments on the potential generalizability of the findings to other settings and that cost effectiveness analysis of this approach is underway. 

 The following are suggestions to clarify aspects of the protocol:

**Background:**

1. While Medellín is clearly highly endemic for dengue, including the historical annual average incidence of dengue would be of interest, as the case-control study will be just be done over a one year time period.  Dengue transmission dropped to unusually low levels throughout most of South America after the Zika epidemic, and is on the increase in the region as of mid-2019.     

**Methods:**

1. The entomologic details regarding the mosquito releases and strain(s) of *Wolbachia* could be expanded upon. What is being done differently in 2018 and 2019 compared to the 2017 releases that achieved temporary high *Wolbachia* prevalence? 
2. More details on the *Wolbachia* sampling and monitoring strategy would be helpful.  What is the extent of the monitoring area?  All comuna outside the case-control area?  Checking BG traps once a week does not ensure that the mosquito specimens will be identified correctly because of damage from the trap fans to captured mosquitoes. Many samples will be lost because of loss of power, ants, etc. BG traps should be checked daily.
3. For the ITS component and the secondary endpoint of severe dengue incidence, how does the Ministry of Health define severe dengue, and has severe dengue been consistently defined over time?
4. Regarding the case control study participant recruitment, what is the possibility that patients with acute febrile illness in the study areas will seek care in clinics outside the network? 
5. With regard to the laboratory testing algorithm to exclude potential dengue serologic positives from being control patients, the authors could more explicitly state that PanBio IgG ELISA test is designed with cutoffs to only detect high IgG titers consistent with acute secondary infections, and not past infections.  Excluding all patients with IgG antibody due to past dengue infection in this setting would eliminate a large percentage of the potential control patients.
6. Consider observing changes in the vector-bacteria-virus interaction through time.  Coevolution is likely. Whether virus blocking ability changes over time (e.g., lowered bacteria concentration in the mosquito cells) is not clear. 

Is the study design appropriate for the research question?

Yes

Is the rationale for, and objectives of, the study clearly described?

Yes

Are sufficient details of the methods provided to allow replication by others?

Partly

Are the datasets clearly presented in a useable and accessible format?

Not applicable

Reviewer Expertise:

Epidemiology, dengue and arbovirology, tropical medicine

We confirm that we have read this submission and believe that we have an appropriate level of expertise to confirm that it is of an acceptable scientific standard.

# Response

**Published:** 2020-05-13

**Authors:** Katherine ANDERS

**Affiliations:** Monash University, Australia

**Background:**

 1. To clarify, the case-control study period was planned to be *a minimum *of 12 months duration but now has funding to continue in order to attain the target sample size, given the low dengue event rate throughout this region of Colombia following a large epidemic in 2016. We have removed the timeline in Figure 4, which is no longer applicable. We have now included the historical annual average incidence of dengue in Medellin and Bello in the background section, as suggested.

**Methods:**

 1.Text has been added to the second paragraph under ‘ *Wolbachia *deployment’ to clarify the changes in *Wolbachia *release material from the 2017 to the 2018/2019 releases, as follows:

 ‘An initial period of *Wolbachia* deployment was undertaken in the early-release zone of the case-control area and in parts of Bello between April and December 2017, resulting initially in a high prevalence of *Wolbachia.* However, *Wolbachia *levels declined after cessation of releases. We believe this was due to fitness issues with the *Wolbachia*-infected mosquitoes, specifically that they were less resistant to insecticides than the wild population. Insectary processes were subsequently updated and a new colony of *Wolbachia *mosquitoes was produced that matched the wildtype insectide resistance profile. Subsequent rounds of deployments were then conducted in these areas between mid-2018 and late-2019. It is this deployment period that will be considered for the purpose of the analyses described in this protocol.’

 2. The text describing the *Wolbachia *sampling and monitoring strategy has been edited to clarify that ‘ *Wolbachia* prevalence is monitored through a network of BG-Sentinel adult mosquito traps (BioGents) that are evenly spaced throughout all of Bello and Medellin, including the case-control study area, at a density of approximately 16 BG traps per km ^2^’, and that ‘ BG traps that do not catch any mosquitoes in three consecutive weeks are moved to another location’. Servicing BG traps more frequently than once a week is not feasible for city-wide projects, and weekly collection is standard across all our project sites. There is certainly the potential for loss of samples due to damage, predators, loss of power etc, but this would result only in a loss of sample size for estimation of *Wolbachia *prevalence, not a bias in these estimates. Trap failures for various reasons are noted at the time of trap servicing and documented in the entomological database. 

 3. The case definition for severe dengue has now been added to the text describing the primary and secondary endpoints for the ITS analysis, as follows:

 ‘Severe dengue is defined as any case of dengue that has one or more of the following manifestations: i) shock or respiratory distress due to severe plasma leakage; ii) severe bleeding according to the evaluation of the treating physician; or iii) severe organ involvement, such as liver damage, impaired consciousness, myocarditis, or other organ involvement.’

 4. We know that a subset of patients with acute febrile illness in the study areas certainly will seek care in clinics outside the network of study clinics, and indeed outside the case-control study area. However the test-negative case-control design is robust to this, based on the assumption that the propensity of patients from the treated and untreated case-control areas to seek care at the network of study clinics is equivalent among test-negative controls (patients with other causes of febrile illness) and test-positive dengue cases. Ie. complete sampling of acute febrile illness patients in the study area is not required, as long as the sampling fraction can be assumed to be equivalent for test-positive cases and test-negative controls. 

 5. This clarification has been added to the text describing methods for laboratory investigations.

 6. Work is ongoing to monitor the co-evolution of *Wolbachia* and *Aegypti* over time in our Australian and international field sites. Preliminary unpublished WMP work has established that the *w*Mel *Wolbachia* genome remains highly stable eight years post-release in northern Australia. Others have shown that pathogen blocking in *Wolbachia*-infected *Ae. aegypti *is stable due to an evolutionary trade off between the mosquito host and the bacteria (Ford et al (2019) *Nature Microbiology*), whereby mutations in *Ae. aegypti *that reduce *Wolbachia*-mediated virus blocking carry a significant fitness cost.
